# Multilocus Sequence Typing helps understand the genetic diversity of *Cryptosporidium hominis* and *Cryptosporidium parvum* isolated from Colombian patients

**DOI:** 10.1371/journal.pone.0270995

**Published:** 2022-07-08

**Authors:** Johanna Uran-Velasquez, Juan F. Alzate, Ana E. Farfan-Garcia, Oscar G. Gomez-Duarte, Larry L. Martinez-Rosado, Diego D. Dominguez-Hernandez, Winston Rojas, Ana Luz Galvan-Diaz, Gisela M. Garcia-Montoya

**Affiliations:** 1 Centro Nacional de Secuenciación Genómica–CNSG, Sede de Investigación Universitaria–SIU, Medellín, Antioquia, Colombia; 2 Departamento de Microbiología y Parasitología, Facultad de Medicina, Universidad de Antioquia, Medellín, Antioquia, Colombia; 3 Grupo Pediaciencias, Facultad de Medicina, Universidad de Antioquia, Medellín, Antioquia, Colombia; 4 Universidad de Santander, Facultad de Ciencias Médicas y de la Salud, Instituto de Investigación Masira, Bucaramanga, Colombia; 5 Department of Pediatrics, Jacobs School of Medicine and Biomedical Sciences, University at Buffalo, State University of New York, Buffalo, NY, United States of America; 6 John R. Oishei Children’s Hospital, Buffalo, NY, United States of America; 7 Equipo Latinoamericano de Investigación en Infectología y Salud Pública (ELISAP), E.S.E. Hospital La María, Medellín, Colombia; 8 Grupo de Investigación en Genética Molecular (GENMOL), Sede de Investigación Universitaria, Universidad de Antioquia, Medellín, Colombia; 9 Grupo de Microbiología Ambiental, Escuela de Microbiología, Universidad de Antioquia, Medellín, Antioquia, Colombia; Midwestern University, UNITED STATES

## Abstract

Multilocus Sequence Typing has become a useful tool for the study of the genetic diversity and population structure of different organisms. In this study, a MLST approach with seven loci (*CP47*, *MS5*, *MS9*, *MSC6-7*, *TP14*, and *gp60*) was used to analyze the genetic diversity of *Cryptosporidium hominis* and *Cryptosporidium parvum* isolated from 28 Colombian patients. Five *Cryptosporidium* species were identified: *C. hominis*, *C. parvum*, *Cryptosporidium felis*, *Cryptosporidium meleagridis*, and *Cryptosporidium suis*. Unilocus *gp60* analysis identified four allelic families for *C. hominis* (Ia, Ib, Id, and Ie) and two for *C. parvum* (IIa and IIc). There was polymorphic behavior of all markers evaluated for both *C. hominis* and *C. parvum*, particularly with the *CP47*, *MS5*, and *gp60* markers. Phylogenetic analysis with consensus sequences (CS) of the markers showed a taxonomic agreement with the results obtained with the *18S rRNA* and *gp60* gene. Additionally, two monophyletic clades that clustered the species *C. hominis* and *C. parvum* were detected, with a higher number of subclades within the monophyletic groups compared to those with the *gp60* gene. Thirteen MLG were identified for *C. hominis* and eight for *C. parvum*. Haplotypic and nucleotide diversity were detected, but only the latter was affected by the *gp60* exclusion from the CS analysis. The gene fixation index showed an evolutionary closeness between the *C. hominis* samples and a less evolutionary closeness and greater sequence divergence in the *C. parvum* samples. Data obtained in this work support the implementation of MLST analysis in the study of the genetic diversity of *Cryptosporidium*, considering the more detailed information that it provides, which may explain some genetic events that with an unilocus approach could not be established. This is the first multilocus analysis of the intra-specific variability of *Cryptosporidium* from humans in South America.

## Introduction

Cryptosporidiosis is an emerging infectious disease of public health significance and environmental challenge worldwide. It is recognized as a major cause of moderate to severe diarrhea, particularly in children and individuals with highly impaired T-cell functions; and waterborne and foodborne outbreaks [1, 2]. *Cryptosporidium* parasites are intracellular microorganisms with a global distribution and a wide range of hosts, including humans. Zoonotic and anthroponotic transmission has been described, mainly through an orofecal route [2]. At least 44 *Cryptosporidium* species and 120 genotypes have been reported until now, and those most identified in humans are *Cryptosporidium hominis* and *Cryptosporidium parvum* [3].

Worldwide studies describe a *Cryptosporidium* prevalence between 1 to 10.7% in children [4, 5], and 8.7% in HIV+ patients [6]. In Colombia, cryptosporidiosis is not a notifiable disease, therefore its frequency may be underestimated. Studies on *Cryptosporidium* infection in children and immunocompromised patients in the country report frequencies between 1.9% and 46.8% [7–9], and 1% and 51.4% [9–11], respectively. Several species have been identified, with most human cases associated with *C*. *hominis* and a lower frequency with *C*. *parvum*, *C*. *meleagridis*, *C*. *felis*, *Cryptosporidium andersoni*, *Cryptosporidium ubiquitum*, *Cryptosporidium muris*, *Cryptosporidium bovis*, and *Cryptosporidium viatorum* [9, 12, 13]. In animals and water sources, species such as *C*. *parvum*, *C*. *hominis*, *Cryptosporidium molnari*, and *Cryptosporidium galli* have also been identified [9, 12, 14].

Unilocus analysis is conventionally used to study the inter and intra-species diversity within *Cryptosporidium* genus, being the *gp60* gene the most frequently employed [15]. However, evidence has increasingly shown the low discriminatory power of this gene as the only marker for the study of *Cryptosporidium* diversity and population structure [16–19].

Multiple loci typing has become one of the most useful strategies to address the genetic diversity or variability of a microorganism such as fungi [20], protists [21], and bacteria [22]. Studying the alleles of different loci increase the resolution power of typing schemes, allowing the detection of genetically distinct lineages. Two main strategies have been developed in multilocus analysis. One is based on the comparative analysis of the size of amplified fragments of multiple loci (Multilocus Fragment Typing-MLFT) and the identification of the length/size of polymorphisms; and a second strategy aimed at the comparative analysis of nucleotide sequences (Multilocus Sequence Typing-MLST), which allows the identification of both length differences and nucleotide variations [17, 23]. However, although MLFT can provide good differentiation and is relatively quick and cheap to perform, MLST is considered the gold standard as it is capable of unequivocally identifying genomic variations, such as single nucleotide polymorphism (SNPs) or point mutations, small insertions and deletions, inversions and translocations, and deletions or duplications [23–26].

Multilocus typing has been a valuable tool in the study of population genetic structure and the transmission dynamics of *Cryptosporidium* [16, 17]. Although MLFT is the most employed strategy, MLST tools are increasingly used for this purpose, mainly for *C*. *parvum* and *C*. *hominis* research [26]. About 50 micro and minisatellite markers have been reported for this purpose, with *CP47*, *gp60*, *MS5*, *MS9*, *MSC 6–7*, *TP14*, *ML2*, *ML1*, *MSB*, and *5B12* loci being the most frequently used [17].

In the present study, species and subtypes of *Cryptosporidium* isolated from 28 Colombian patients were detected through the sequence analysis of partial regions of the *18S rRNA* and *gp60* genes, respectively. In addition, a multi-locus genotyping study was performed through the analysis of the *CP47*, *gp60*, *ML2*, *MS5*, *MS9*, *MSC6–7*, and *TP14* loci. Phylogenetic analyses were performed with the CS of the loci evaluated, and the Multilocus Genotypes (MLG) and genetic diversity indices were obtained.

## Material and methods

### Sample collection and DNA extraction

A total of 28 stool samples positive for *Cryptosporidium* by PCR of the *18S rRNA* gene were included. Twenty samples came from school-age children from two Colombian provinces (Antioquia and Santander) [13, 27], and eight from HIV-positive patients from Antioquia province. Diarrhea was reported in 55.5% of the children, and all the HIV + patients. Total DNA was extracted from the samples using the Norgen Stool DNA Isolation Kit (Thorold, Canada), and following the manufacturer’s recommendations. DNA obtained was quantified by absorption at 260nm (NANODROP 2000c Thermo Scientific; Waltham, USA), labeled and stored at -30°C for subsequent analysis.

### Species identification and *gp60* subtyping

For species identification a nested PCR that amplify a fragment of approximately 830 bp of the *Cryptosporidium 18S rRNA* gene was done following the protocol by Xiao et al. [28]. All PCR analyses included positive and negative controls. They were also tested for bacterial *16S rDNA* gene using 27F and 1492R primers [29] to rule out PCR inhibitors and DNA instability. The amplified products were used for the phylogenetic analysis. A dataset was constructed with the refined and assembled nucleotide sequences corresponding to the 28 samples plus the reference sequences of the *18S rRNA* gene of 16 *Cryptosporidium* species deposited in the GenBank (S1 File). *Eimeria tenella* (U40264.1) was used as an outgroup.

For *Cryptosporidium* subtyping, a nested PCR of the *gp60* gene was performed on the samples where the species *C*. *hominis*, *C*. *parvum*, and *C*. *meleagridis* were previously identified. For the first two species, a fragment of approximately 850 bp was obtained following the protocol described by Alves et al. [30]. For *C*. *meleagridis*, a fragment of 900 bp was amplified according to the protocol described by Stensvold et al. [31]. The amplified products were sequenced, and two datasets were used to construct a phylogenetic tree; the first one was constructed with the nucleotide sequences of the parasites identified as *C*. *hominis* and *C*. *parvum* in the present work, plus 29 reference sequences of the *gp60* gene for *C*. *hominis*; and 18 *C*. *parvum* sequences (S1 File). *Cryptosporidium meleagridis* (AF401499) was used as an outgroup. The second dataset was built with the nucleotide sequences of the sample identified in this work as *C*. *meleagridis*, plus 32 reference sequences of the *gp60* gene for this species (S1 File). *Cryptosporidium parvum* (AY262034) was used as an outgroup.

### *In silico* validation of different gene loci

**Fig 1:**
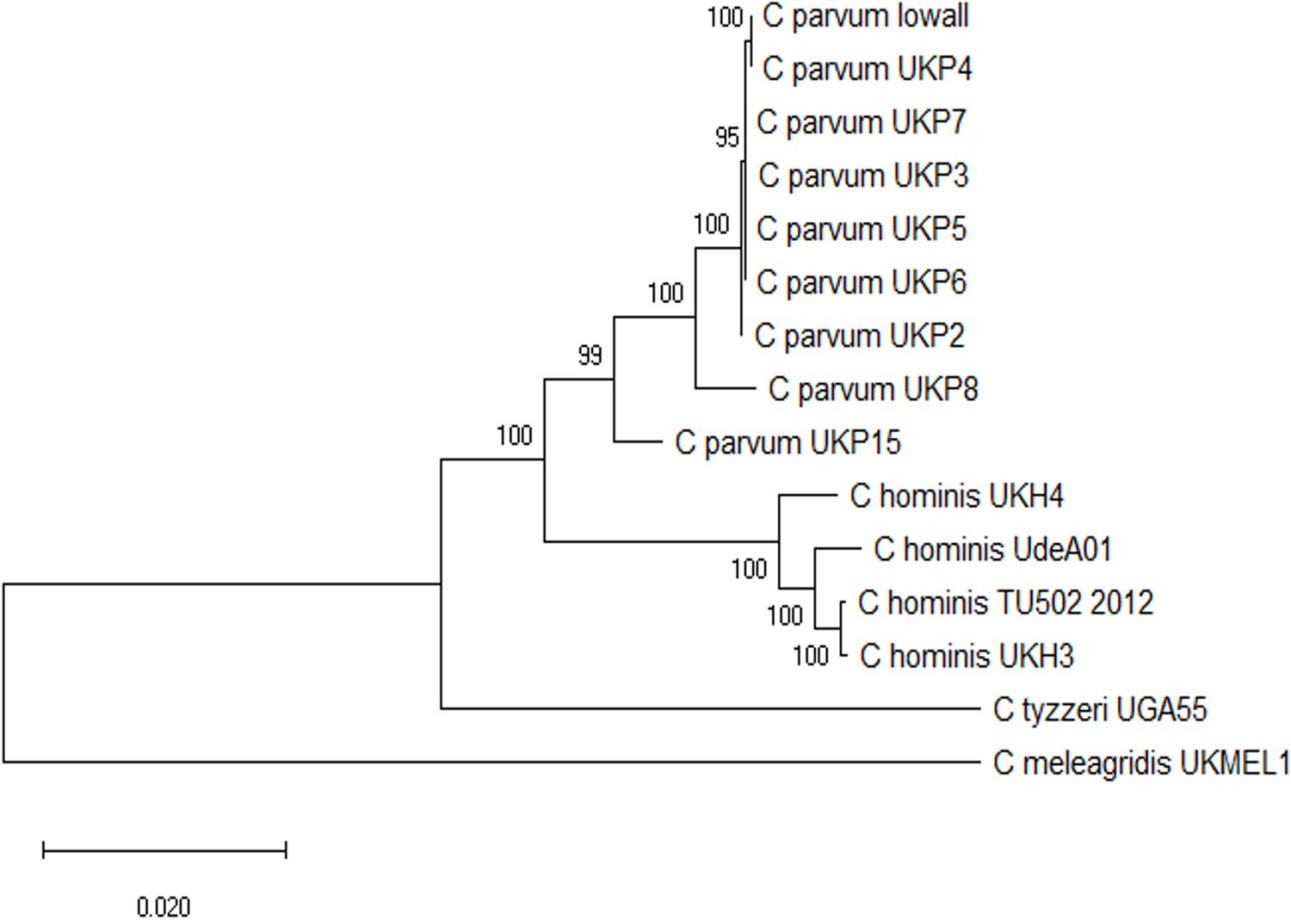
Phylogenetic analysis with the CS of the seven markers in the *Cryptosporidium* genomes used for the validation strategy.

Since a MLST analysis protocol for *Cryptosporidium* has not yet been standardized, in our study an initial validation strategy for the sequences of seven loci (*CP47*, *gp60*, *MS5*, *MS9*, *MSC6*–*7*, *TP14*, and *ML2*) was done. Each marker was searched on the genomes from different *Cryptosporidium* species. Five genomes were selected from CryptoDB (https://cryptodb.org/cryptodb/app), and 10 from the Sequence Read Archive (SRA) (https://www.ncbi.nlm.nih.gov/sra) databases from the NCBI. Selection parameters included a nucleotide identity greater than 80% and E = 1e-20 value for each of the sequences of the seven loci. The taxonomic resolution power of the selected markers was tested using a concatenated alignment strategy. A maximum-likelihood tree was constructed with the 15 CS. Phylogenetic analysis with CS showed two main clades, one that clusters the *C*. *hominis* genomes, and in the other, those of *C*. *parvum*; *Cryptosporidium tyzzeri* and *C*. *meleagridis* genomes were separated from the rest (Fig 1; S1 File).

Phylogenetic relationships between the 15 CS constructed with the seven markers (*CP47*, *gp60*, *ML2*, *MS5*, *MS9*, *MSC6*–*7*, and *TP14*) from the genomes used for *in silico* analysis, was inferred by using the Maximum Likelihood method and General Time Reversible model. A discrete Gamma distribution was used to model evolutionary rate differences among sites. The tree is drawn to scale, with branch lengths measured in the number of substitutions per site. Bootstrap values greater than 50% from 1,000 replicates are shown.

### Unilocus analysis using different loci on *C*. *hominis* and *C*. *parvum* samples

PCR protocols for each genetic marker were standardized (S1 File). Six samples and the *ML2* gene were excluded from the study. Unilocus analysis with each marker (*CP47*, *gp60*, *MS5*, *MS9*, *MSC6–7*, and *TP14)* was performed in 22 samples (14 for *C*. *hominis*, and 8 for *C*. *parvum*). A dataset with the sequences obtained was built with the information of each marker and the 22 samples. The sequences from the *C*. *hominis* (TU502-2012 and UKH3) and *C*. *parvum* (Iowa II, UKP2, UKP5, UKP6, UKP7, UKP8, and UKP15) genomes were used as a reference. The sequences obtained from the *C*. *meleagridis* UKMEL1 genome were used as an outgroup. Refined sequences were also employed for the nucleotide comparisons of each marker per sample using the DNAsp program (http://www.ub.edu/dnasp/). The differences found within each marker in the *C*. *hominis* and *C*. *parvum* samples allowed the generation of the different alleles. All analyses were carried out with the statistical program R-Studio (http://www.rstudio.com/).

### Sequence and phylogenetic analyses

PCR primers, reagents, and cycling conditions are described in the supplementary material (S1 File). The amplified products were sent to the MACROGEN company (Seoul, Korea) for capillary sequencing in both strands. All the sequences for the unilocus and multilocus analysis were aligned with the MAFFT program version 7 (https://mafft.cbrc.jp/alignment/software/), and the phylogeny was inferred using the MEGA program version 7 (http://www.megasoftware.net/). The sequences from the study were submitted to GenBank (S1 File).

### MLST with the CS of different loci on *C*. *hominis* and *C*. *parvum* samples

CS was constructed with the six previously selected loci (*CP47*, *gp60*, *MS5*, *MS9*, *MSC6–7*, and *TP14*) and each sample. Then, this dataset, including two references of *C*. *hominis* (TU502-2012 and UKH3) and seven of *C*. *parvum* (Iowa II, UKP2, UKP5, UKP6, UKP7, UKP8, and UKP15), were employed for the reconstruction of a maximum-likelihood tree. The CS of the six markers obtained from the *C*. *meleagridis* isolate (UKMEL1) was used as an outgroup. Subsequently, CS was used to identify the MLG using the DNAsp program (http://www.ub.edu/dnasp/), and the Network program version 4.6.1.0 for the generation of a haplotype network graphic.

A dataset of the CS corresponding to the identified MLG was used to analyze the genetic diversity of the *C*. *hominis* and *C*. *parvum* samples, using the DNAsp program (http://www.ub.edu/dnasp/). The following statistical analyses were done: Haplotype diversity (Hd); number of segregating sites (S); nucleotide diversity (Pi); and average number of nucleotide differences (K). These analyses were carried out intra-species (*C*. *hominis* or *C*. *parvum*) and among the population evaluated (children and HIV- positive patients) within each species. The gene fixation index (Fst) was estimated using the Arlequin version 3.5 program (http://cmpg.unibe.ch/software/arlequin35). Analyses were performed with the CS with and without the information corresponding to the *gp60* gene to evaluate the contribution of this marker in the statistical data obtained.

### Ethical considerations

The study protocol followed the ethical guidelines of the 2013 Declaration of Helsinki. All ethical approvals for the study were obtained according to the ethical standards set by the Ministry of Health of Colombia (Resolution 008430 of 1993). All the samples included in this work were anonymized and collected in previous research projects, in which informed consent documents were signed by the parents or legally authorized representatives of the children and by the HIV patients included in the study. The Bioethics Committee from Sede de Investigación Universitaria-SIU, Universidad de Antioquia (official document N° 20-06-913); and the institutional review boards (IRB) at the University of Buffalo, Vanderbilt University and Universidad de Santander approved the investigation protocol.

## Results

### Species identification and *gp60* subtyping

**Fig 2:**
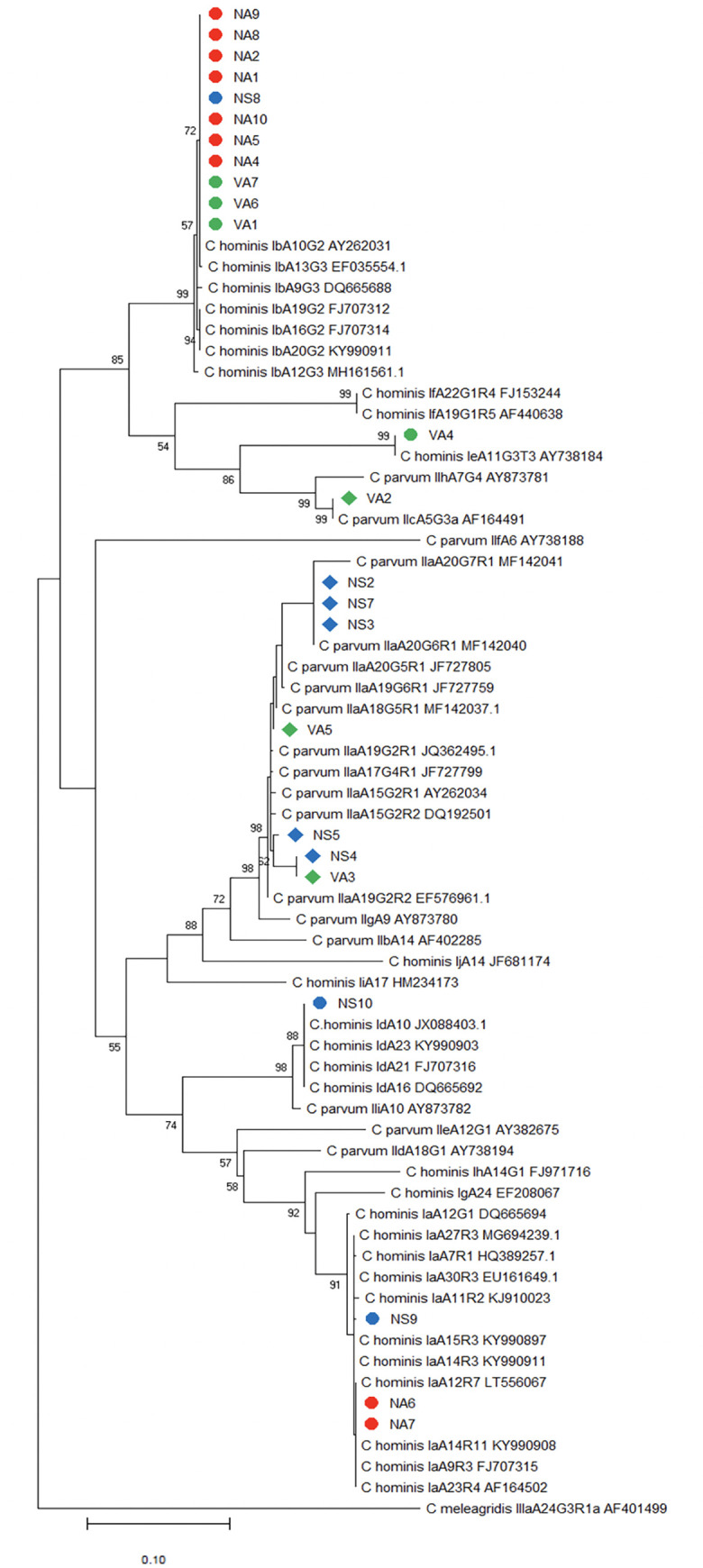
Phylogenetic relationships of *Cryptosporidium hominis and C*. *parvum* at the *gp60* locus.

**Fig 3:**
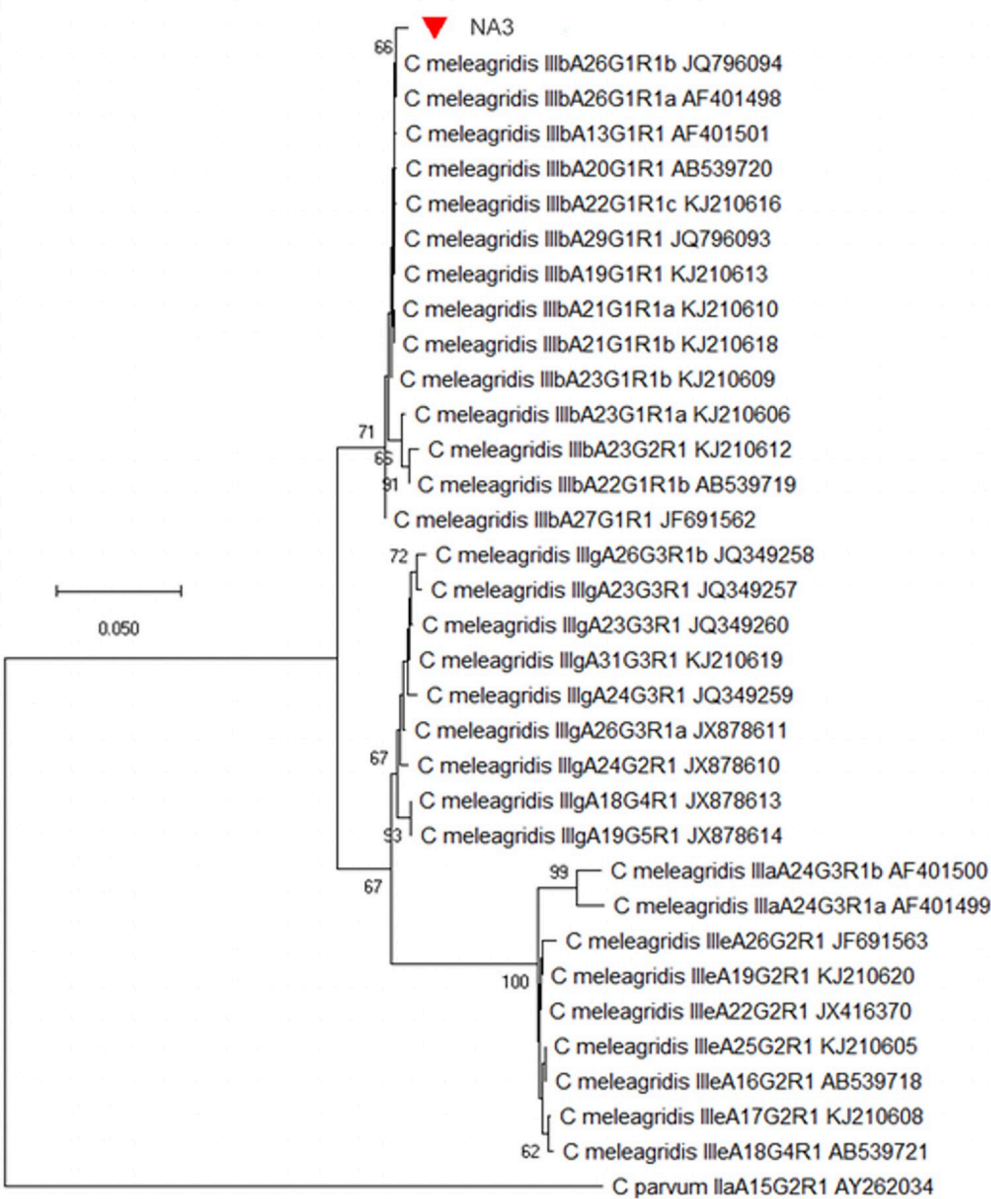
Phylogenetic relationships of *Cryptosporidium meleagridis* at the *gp60* locus.

Five *Cryptosporidium* species were identified in 28 samples from Colombian patients. *Cryptosporidium hominis* was detected in 57.1% (16/28; 12 children and 4 HIV patients) of the samples; *C*. *parvum* in 28.6% (8/28; 5 children and 3 HIV patients); *C*. *felis* in 7.1% (2/28; one child and one HIV patient); and both *C*. *meleagridis* and *C*. *suis* in 3.6% (1/28; one child) (Table 1). *Cryptosporidium hominis* IbA10G2 *gp60* subtype was identified in 11/16 samples (68.8%), being the most common; IaA13R7, IaA11R3, IaA13R6, IdA10, and IeA11G3T3 subtypes were identified in one sample (Fig 2, Table 1). *Cryptosporidium parvum* IIaA20G6R1 (3/8; 37.5%) and IIaA19G5R1 (2/8; 25%) *gp60* subtypes were the most frequently detected; IIaA18G5R1, IIaA17G4R1, and IIcA5G3a subtypes were identified in one sample (Fig 2, Table 1). *Cryptosporidium meleagridis* IIIbA26G1R1 *gp60* subtype was confirmed (Fig 3, Table 1).

**Table 1:** Species identification and *gp60* unilocus genotype.

*Cryptosporidium* species	Species frequency %	Subgenotype *gp60*	Subgenotype frequency %	School-age children from two Colombian provinces		HIV (+) patients from Antioquia VA (n = 8)
				Antioquia NA (n = 10)	Santander NS (n = 10)	
***C*. *hominis***	**16/28 (57.1%)**	**IbA10G2**	11/16 (68.8%)	n = 7 NA: 1,2,4,5,8–10	n = 1 NS8	n = 3 VA: 1, 6,7
		*IaA13R6	1/16 (6.3%)	n = 1 NA6	-	-
		**IaA13R7**	1/16 (6.3%)	n = 1 NA7	-	-
		*IaA11R3	1/16 (6.3%)	-	n = 1 NS9	-
		**IeA11G3T3**	1/16 (6.3%)	-		n = 1 VA4
		**IdA10**	1/16 (6.3%)	-	n = 1 NS10	-
***C*. *parvum***	**8/28 (28.6%)**	**IIaA20G6R1**	3/8 (37.5%)	-	n = 3 NS: 2,3,7	-
		*IIaA19G5R1	2/8 (25%)	-	n = 1 NS4	n = 1 VA3
		**IIaA18G5R1**	1/8 (12.5%)	-	n = 1 NS5	-
		**IIaA17G4R1**	1/8 (12.5%)	-	-	n = 1 VA5
		**IIcA5G3a**	1/8 (12.5%)	-	-	n = 1 VA2
***C*. *meleagridis***	**1/28 (3.6%)**	**IIIbA26G1R1**	1/1 (100%)	n = 1 NA3	-	-
***C*. *felis* 2/28 (7.1%)**		-			n = 1 NS1	n = 1 VA8
* ***C*. *suis***	**1/28 (3.6%)**	-			n = 1 NS6	-
**Total**		**-**		**10**	**10**	**8**

NA: Antioquia children.

NS: Santander children.

VA: Antioquia HIV+ individuals.

* First report in Colombia.

The evolutionary history was inferred by using the Maximum Likelihood method and Tamura-Nei model. A discrete Gamma distribution was used to model evolutionary rate differences among sites. The tree is drawn to scale, with branch lengths measured in the number of substitutions per site. Bootstrap values greater than 50% from 10,000 replicates are shown. The sequence *gp60* from *C*. *melagridis* IIIa AF401499 was used as an outgroup. Evolutionary analyses were conducted in MEGA7. The circle symbol corresponds to *C*. *hominis*, the rhombus to *C*. *parvum* and the red, blue and green color to children from Antioquia, Santander and HIV positive patients, respectively.

The evolutionary history was inferred by using the Maximum Likelihood method and Tamura-Nei model. A discrete Gamma distribution was used to model evolutionary rate differences among sites. The tree is drawn to scale, with branch lengths measured in the number of substitutions per site. Bootstrap values greater than 50% from 10,000 replicates are shown. The sequence *gp60* from *C*. *parvum* II A4262034 was used as an outgroup. Evolutionary analyses were conducted in MEGA7. The rhombus red symbol corresponds to *C*. *meleagridis* to children from Antioquia.

### Unilocus analysis with *CP47*, *gp60*, *MS5*, *MS9*, *MSC6–7*, and *TP14* loci in the *C*. *hominis* and *C*. *parvum* samples

**Fig 4:**
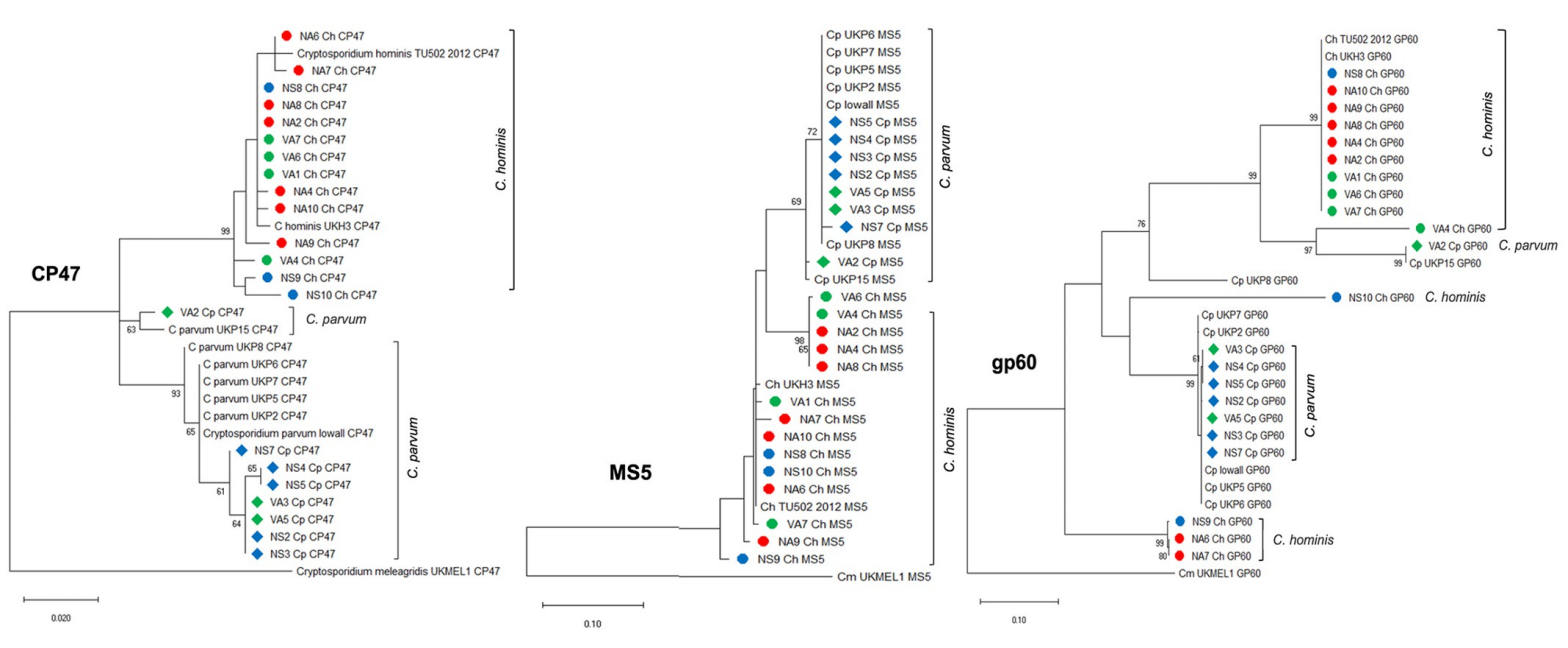
Phylogenetic trees constructed with the sequences of *CP47*, *gp60*, *MS5* markers genes.

**Fig 5:**
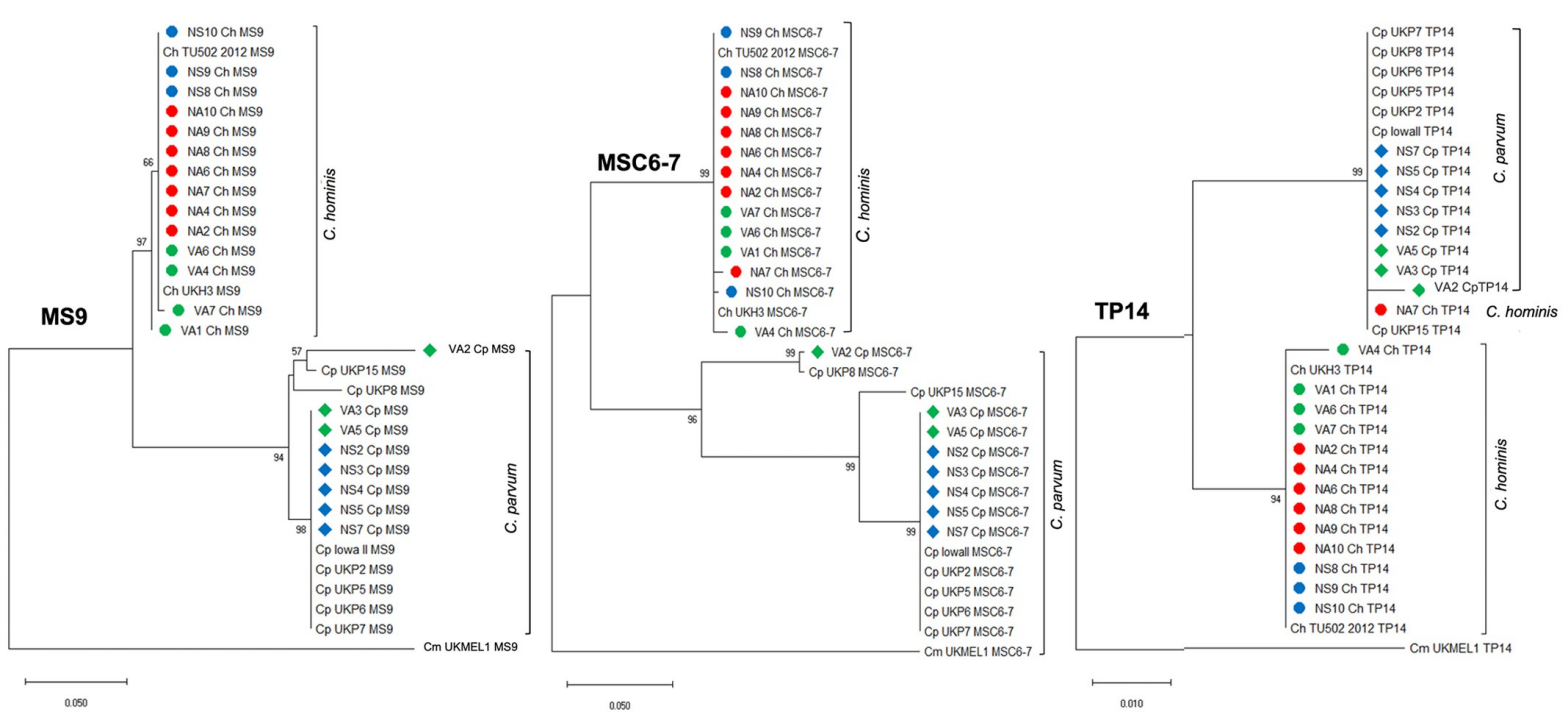
Phylogenetic trees constructed with the sequences of *MS9*, *MSC6*–*7*, and *TP14* markers genes.

Phylogenetic trees for each of the six loci and 22 samples evaluated were constructed (Figs 4 and 5), and the alleles were identified (Table 2). These markers showed a separation of *C*. *hominis* and *C*. *parvum* samples into two clades (Figs 4 and 5), *CP47*, *MS5*, *MS9*, *MSC6–7*, and *TP14* loci perform better than the *gp60* gene for clustering and separation of these species. The *CP47* marker had the highest number of intra-species subclades for both *C*. *hominis* and *C*. *parvum* (Fig 4).

**Table 2:** Allele identification for *CP47*, *gp60*, *MS5*, *MS9*, *MSC6*–*7*, and *TP14* in *C*. *hominis* and *C*. *parvum* positive samples.

***C*. *hominis***		**Markers with micro/minisatellite sequences**					
**Host**	**Sample**	***CP47* Allele**	***gp60* Allele / Subgenotype**	***MS5* Allele**	***MS9* Allele**	***MSC6***–***7* Allele**	***TP14* Allele**
Children from Antioquia **n = 7**	NA2	1	1 / IbA10G2	1	1	1	1
	NA4	2	1 / IbA10G2	1	1	1	1
	NA6	3	2 / IaA13R6	2	2	2	2
	NA7	4	3 / IaA13R7	3	1	3	3
	NA8	1	1 / IbA10G2	1	1	1	1
	NA9	5	1 / IbA10G2	4	1	2	4
	NA10	6	1 / IbA10G2	5	1	2	4
Children from Santander **n = 3**	NS8	1	1 / IbA10G2	6	1	2	4
	NS9	7	4 / IaA11R3	7	1	2	4
	NS10	8	5 / IdA10	5	1	4	5
HIV (+) patients from Antioquia **n = 4**	VA1	1	1 / IbA10G2	8	3	2	4
	VA4	9	6 / IeA11G3T3	1	1	5	6
	VA6	1	1 / IbA10G2	9	1	1	1
	VA7	1	1 / IbA10G2	10	4	2	4
**Total allele number**		**9**	**6**	**10**	**4**	**5**	**6**
***C*. *parvum***		**Markers with micro/minisatellite sequences**					
**Host**	**Sample**	***CP47* Allele**	***gp60* Allele / Subgenotype**	***MS5* Allele**	***MS9* Allele**	***MSC6***–***7* Allele**	***TP14* Allele**
Children from Santander **n = 5**	NS2	1	1 / IIaA20G6R1	1	1	1	1
	NS3	2	1 / IaA20G6R1	1	1	1	2
	NS4	3	2 / IIaA19G5R1	1	1	2	1
	NS5	4	3 / IIaA18G5R1	1	2	1	3
	NS7	5	1 / IIaA20G6R1	2	1	1	1
HIV+ patients from Antioquia **n = 3**	VA2	6	4 / IIcA5G3a	3	3	3	4
	VA3	1	2 / IIaA19G5R1	4	4	1	1
	VA5	7	5 / IIaA17G4R1	4	1	1	5
**Total allele number**		**7**	**5**	**4**	**4**	**3**	**5**

NA: Antioquia children.

NS: Santander children.

VA: Antioquia HIV+ individuals.

* First report in Colombia.

Phylogenetic relationships between the sequences of the six markers in *C*. *hominis* (14) and *C*. *parvum* (8) samples with *CP47*, *gp60*, and *MS5*. The evolutionary history was inferred by using the Maximum Likelihood method based on the Hasegawa-Kishino-Yano model for *CP47* and *MS5*; and for *gp60* the Tamura-Nei model with a discrete Gamma distribution to model evolutionary rate differences among sites. The sequence corresponding to each marker obtained from the *C*. *meleagridis* UKMEL1 genome was used as an outgroup. The trees are drawn to scale, with branch lengths measured in the number of substitutions per site. Bootstrap values greater than 50% from 10,000 replicates are shown. Evolutionary analyses were conducted in MEGA7. The circle symbol corresponds to *C*. *hominis*, the rhombus to *C*. *parvum* and the red, blue and green color to children from Antioquia, Santander and HIV positive patients, respectively.

Phylogenetic relationships between the sequences of the six markers in *C*. *hominis* (14) and *C*. *parvum* (8) samples with *MS9*, *MSC6*–*7*, and *TP14*. The evolutionary history was inferred by using the Maximum Likelihood method based on the Hasegawa-Kishino-Yano model for *MS9* and *TP14*; and Tamura 3 parameter for *MSC6*–*7*. The sequence corresponding to each marker obtained from the *C*. *meleagridis* UKMEL1 genome was used as an outgroup. The trees are drawn to scale, with branch lengths measured in the number of substitutions per site. Bootstrap values greater than 50% from 10,000 replicates are shown. Evolutionary analyses were conducted in MEGA7. The circle symbol corresponds to *C*. *hominis*, the rhombus to *C*. *parvum* and the red, blue and green color to children from Antioquia, Santander and HIV positive patients, respectively.

*Cryptosporidium hominis* positive samples (n:14) showed a polymorphic nature with the six loci evaluated, *CP47* and *MS5* being the most polymorphic (9 and 10 alleles, respectively). Two samples (NA2 and NA8) showed the same allelic profile with all the loci (Table 2), and they were in the same clade in the phylogenetic trees constructed for each marker (Fig 4). In three samples (NA6, NA7, and NS9) we observed unique alleles with *CP47*, *gp60*, and *MS5* loci (Table 2).

*Cryptosporidium parvum* samples (n: 8) were separated into two clades, one with one sample (VA2) and the rest in the second clade. This result was observed with all the loci evaluated. Only the VA2 sample showed a unique profile and different alleles in all the markers (Table 2; Figs 4 and 5). *CP47* was also the most polymorphic marker for this species.

### MLST with *CP47*, *gp60*, *MS5*, *MS9*, *MSC6–7*, and *TP14* loci in the *C*. *hominis* and *C*. *parvum* samples

**Fig 6:**
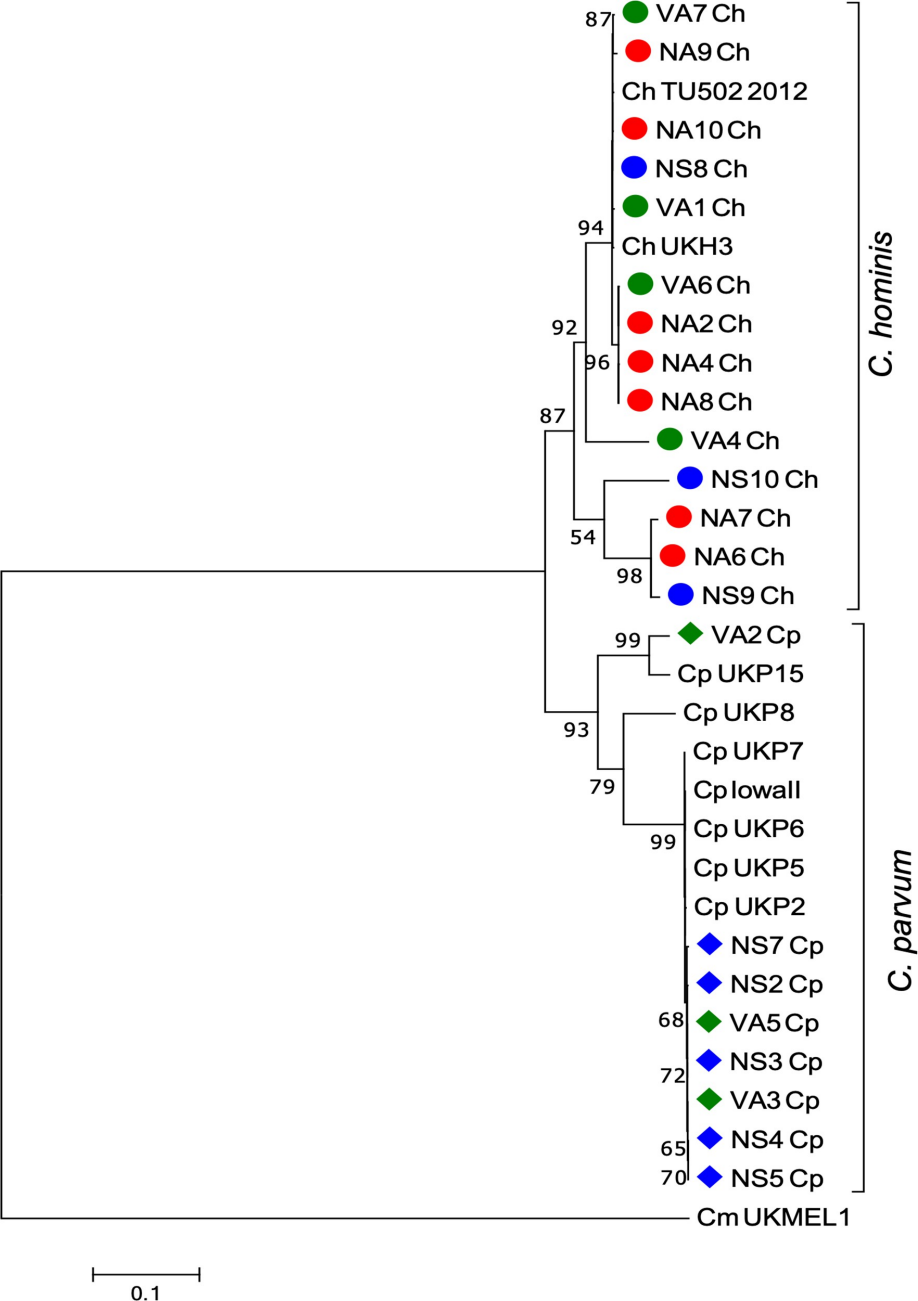
Phylogenetic analysis with the CS of the six markers for the 22 samples from Colombian patients identified as *C*. *hominis* and *C*. *parvum*.

MLST analyses using the 22 CS of the six markers showed the formation of two monophyletic groups corresponding to the *C*. *hominis* and *C*. *parvum* species, with high bootstrap values for each clade (Fig 6). *Cryptosporidium hominis* samples were included in different intra-species subclades. Three samples (NA6, NA7, and NS9) were classified in the Ia *gp60* subtype family and were included in the same subclade. NS10 (Id *gp60* subtype family) and VA4 (Ie *gp60* subtype family) samples were separated in different intra-species subclades (Fig 6, Table 2). The remaining *C*. *hominis* samples (NA1, NA2, NA4, NA 6–9, NA10, and NS8) were in the same subclade and showed the same *gp60* subtype (IbA10G2). Most of the samples had different allelic profiles based on the *CP47*, *gp60* (allelic family Ia), and *MS5* markers analysis. Two samples (NA2 and NA8) had the same allelic profile in all loci (Fig 6, Table 2). The formation of intra-species subclades and evidence of polyphyletic groups between them could be an effect of the nucleotide variations or differences found mainly in the three most polymorphic markers (*CP47*, *MS5*, and *gp60*) (Figs 4 and 6; Table 2).

Phylogenetic relationships between the CS of the six markers for each of the fourteen *C*. *hominis* samples and the eight *C*. *parvum* samples. The CS of the *C*. *hominis* TU502-2012 and UKH3 genomes was used as a reference. For *C*. *parvum*, UKP2, UKP2, UKP5, UKP6, UKP7, UKP15 and Iowa II genomes were used. The evolutionary history was inferred by using the Maximum Likelihood method based on the Tamura-Nei model. A discrete Gamma distribution was used to model evolutionary rate differences among sites. The rate variation model allowed for some sites to be evolutionarily invariable. The tree is drawn to scale, with branch lengths measured in the number of substitutions per site. Bootstrap values greater than 50% from 10,000 replicates are shown. *C*. *meleagridis* UKMEL1 was used as an outgroup. Evolutionary analyses were conducted in MEGA7. The circle symbol corresponds to *C*. *hominis*, the rhombus to *C*. *parvum* and the red, blue, and green color to children from Antioquia, Santander and HIV positive patients respectively.

Concerning *C*. *parvum*, two intra-species subclades were observed, in one of them only the VA2 sample (IIc *gp60* subtype family) was included, which had a unique allelic profile. The remaining samples were grouped in the same clade. All were classified into the IIa *gp60* subtype family but showed allelic differences with the other markers (Fig 6, Table 2).

### *C*. *hominis* and *C*. *parvum* MLG

Thirteen MLG were identified for *C*. *hominis* and eight for *C*. *parvum* (Table 3). The nucleotide variations that led to the 21 MLG designation among the 22 samples were mainly due to tandem repeats of the micro/minisatellite sequences. However, single nucleotide polymorphisms (SNPs) were detected in areas other than those of the repetitive region, particularly in the *CP47*, *gp60*, and *MS5* loci. In this analysis, only two *C*. *hominis* positive samples, both from children, showed the same MLG (NA2 and NA8). Additionally, the number of MLG detected was higher compared to the *gp60* subtypes identified in these samples (Tables 2 and 3). It was also observed that the NA4, NS8, VA6 (*C*. *hominis*), and VA3 (*C*. *parvum*) samples had a difference in the CS in only one of the markers, which was sufficient for the identification of a particular MLG (Tables 1 and 3).

**Table 3:** MLG based on the CS for *C*. *hominis* and *C*. *parvum* parasites identified in Colombian samples.

***C*. *hominis* samples**		
**Sample**	**MLG**	***gp60* subtype**
NA2	MLG1	IbA10G2
NA4	MLG2	IbA10G2
NA6	MLG3	IaA13R6
NA7	MLG4	IaA13R7
NA8	MLG1	IbA10G2
NA9	MLG5	IbA10G2
AC10	MLG6	IbA10G2
NS8	MLG7	IbA10G2
NS9	MLG8	IaA11R3
NS10	MLG9	IdA10
VA1	MLG10	IbA10G2
VA4	MLG11	IeA11G3T3
VA6	MLG12	IbA10G2
VA7	MLG13	IbA10G2
**n: 14**	**Total: 13**	**Total: 6**
***C*. *parvum* samples**		
**Sample**	**MLG**	***gp60* subtype**
NS2	MLG4*	IIaA20G6R1
NS3	MLG5*	IIaA20G6R1
NS4	MLG6*	IIaA19G5R1
NS5	MLG7*	IIaA18G5R1
NS7	MLG8*	IIaA20G6R1
VA2	MLG1*	IIcA5G3a
VA3	MLG2*	IIaA19G5R1
VA5	MLG3*	IIaA17G4R1
**n: 8**	**Total: 8**	**Total: 5**

**Note:** Children Antioquia: NA (2,4,6–10). Santander children: NS (2–5, 7–10). HIV (+) patients Antioquia: VA (1–7). MLG corresponding to the species *C*. *parvum* are marked with an asterisk (*).

### MLG network

**Fig 7:**
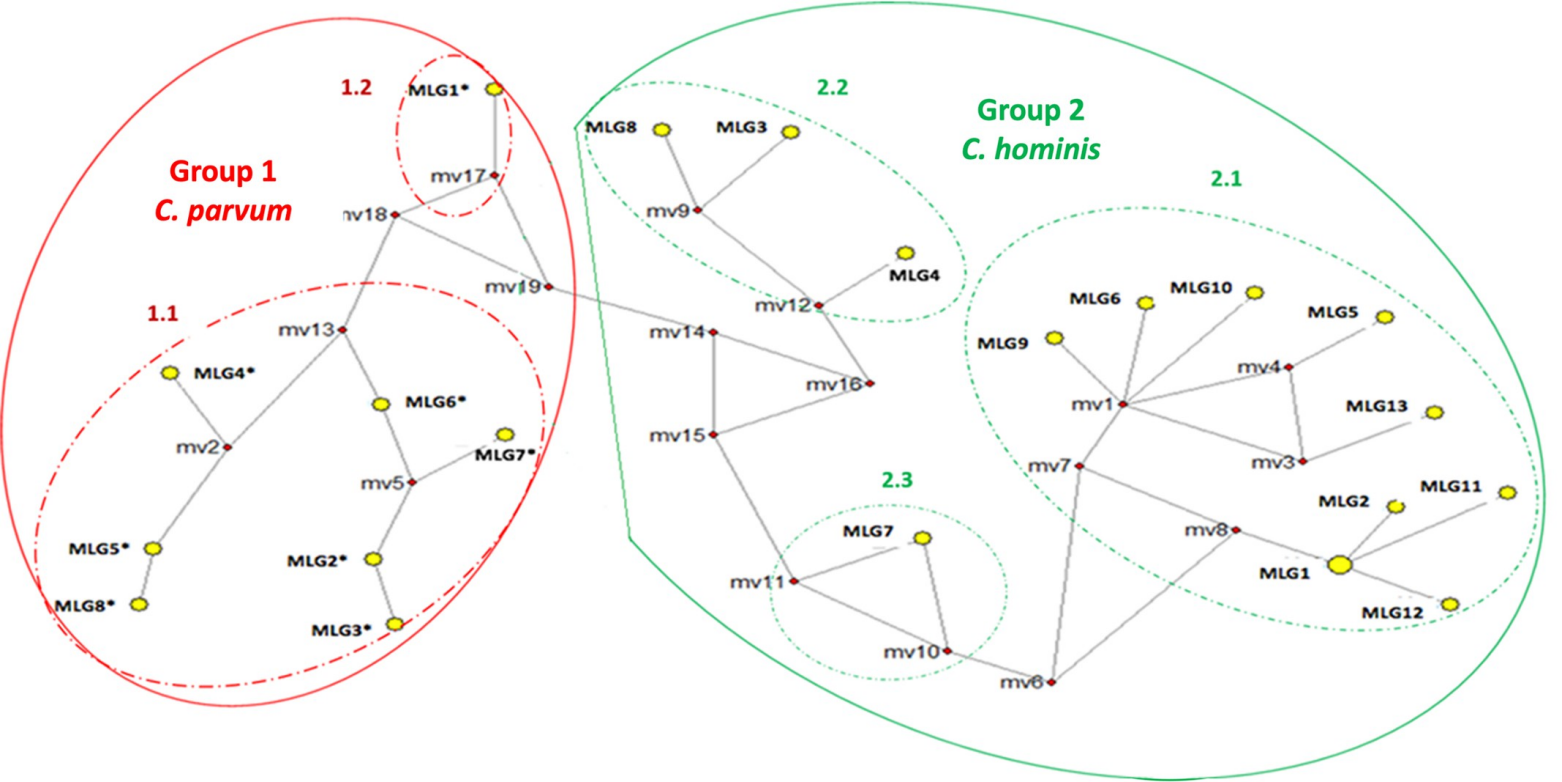
MLG Network constructed with the CS of the six markers for the *C*. *hominis* and *C*. *parvum* samples.

MLG network analysis showed de formation of two groups: one that includes all the *C*. *parvum* samples (MLG 1–8 *), and another with all the *C*. *hominis* samples (MLG 1–13) (Fig 7). *Cryptosporidium parvum* group showed two internal subgroups. In one of them, seven MLG were included (NS 2–5; NS7, VA3, and VA5 samples), while in the second, there was only the MLG corresponding to the VA2 sample. This *C*. *parvum* positive sample has shown a unique and different behavior compared with the other samples in the different analyses carried out in the study.

MLG Network built with the CS of *CP47*, *gp60*, *MS5*, *MS9*, *MSC6*–*7*, and *TP14* markers in the 22 *Cryptosporidium* positive samples from Colombian patients. The Network program (version 10.0) and the median-joining algorithm were used. The graph shows small, red-colored diamonds representing the hypothetical haplotypes (mv), solid, yellow-colored circles representing the different MLG identified in the clinical study populations (their size is proportional to the frequency of the MLG in the population). Large continuous red and green circles cluster the two main groups, and the dashed circles the inner groups. Table 3 shows the sample for which each of the MLG corresponds. The MLG marked with an asterisk "*" correspond to the *C*. *parvum* samples.

In the *C*. *hominis* group there was a separation of the NA6, NA7, and NS9 samples, which corresponded to MLG 3, 4, and 8, respectively (Fig 7). This data could be explained by the nucleotide sequences differences in the CS of the three most polymorphic markers (*CP47*, *gp60*, and *MS5*), showing unique alleles in these samples (Table 2). Regarding MLG7, which corresponded to the NS8 sample, there was a unique sequence for the *MS5* marker, with a lower number of minisatellite tandem repeats compared with the other samples of the same group (Fig 7). Finally, in the distribution analysis of the MLG in this group, MLG1 (NA2 and NA8) was identified as an ancestral MLG, as it was the most frequent and gave rise to other MLG (2, 11, and 12), corresponding to NA4, VA4, and VA6 samples, respectively (Fig 7).

The CS dataset of the identified MLG was statistically analyzed to determine the genetic diversity observed in both *C*. *hominis* and *C*. *parvum*. Haplotype diversity was observed in both species, which could be associated with the number of MLG found (13 MLG in *C*. *hominis* and 8 MLG in *C*. *parvum*). However, higher Pi data was found among the *C*. *hominis* samples compared to *C*. *parvum* samples, indicating a greater nucleotide diversity in the former species (S1 File). A higher number of segregating sites (S) and a higher average number of nucleotide differences (K) were also observed in the *C*. *hominis* samples compared with *C*. *parvum*. For both *C*. *hominis* and *C*. *parvum* samples, when the analyses were performed excluding *gp60* from CS, the number of MLG and Hd remained the same, but the Pi, S, and K values decreased (S1 File).

The gene fixation index (Fst) for the comparisons between the clinical populations (children and HIV-positive patients) showed that within the *C*. *hominis* samples, this value was low and without statistical significance, suggesting that these populations are related, with evolutionary closeness or a recent common ancestor. Both populations showed low genetic differentiation and shared diversity. Moreover, comparisons between children and HIV-positive patients in *C*. *parvum* revealed gene differentiation in the paired comparison (Fst: 0.23; p = 0.018), which indicated a lower evolutionary closeness; it also showed the highest sequence divergence, which seemed to be determined mainly by the VA2 sample. When this value was determined for both species excluding *gp60* from the CS, similar results were obtained to those observed when this marker was included (Fst: 0.24; p = 0.01) (S1 File).

## Discussion

In this work, we present genotyping and genetic variability data on the *Cryptosporidium* genus detected in stool samples from Colombian patients (school-age children and HIV positive adults). Novel circulating species (*C*. *suis*) and subtypes (IaA13R6, IaA11R3, and IIaA19G5R1) were identified in the country. Although our data suggest a predominance of an anthroponotic transmission route, which has been described for *C*. *hominis* and *C*. *parvum* (particularly IIc subtype family) in low- and middle-income countries [32], the circulation of other species also confirm a *Cryptosporidium* zoonotic transmission in the studied areas.

Genetic diversity was evaluated with the *gp60* locus and other genetic markers (*CP47*, *MS5*, *MS9*, *MSC6–7*, and *TP14)*, being *CP47* and *MS5* the most polymorphic, followed by *gp60* and *TP14*. Our findings agree with other studies conducted on *C*. *hominis* and *C*. *parvum* isolates from humans and animals, showing *CP47* as a highly polymorphic marker, which support the use of this loci as a good alternative for *C*. *hominis* and *C*. *parvum* inter and intraspecies genotyping [33–37]. Regarding *MS5*, our results are supported by prior investigations that report a polymorphism among human samples, while in cattle, a monomorphic and polymorphic behavior have been described [16, 38–41].

Our MLST analysis detected a *gp60* intra-subtype variation in both *C*. *parvum* and *C*. *hominis* samples, confirming that the unilocus approach underestimates the genetic diversity of the parasite, which has also been reported by other authors [19, 24]. It seems that *gp60* evolutionary changes occurred earlier than in other genes, and probably even before the differentiation of the species [19, 24], which could explain the variations identified with other markers within *gp60* subtypes. Other multilocus studies carried out in *C*. *hominis*, *C parvum*, and other species such as *C*. *meleagridis*, *Cryptosporidium cuniculus* and *C*. *ubiquitum* have also demonstrated a separation of lineages influenced by the *gp60* subtype, but with differences in other markers that reflect genetic variability associated with evolutionary relationships [42–44]. Nader et al. described that genes with a positive selection and probably related to virulence factors, including *gp60* and *CP47*, are in the genome “recombinant” regions, indicating an adaptive genetic introgression or gene flow between *Cryptosporidium* subtypes and species, which could occur over a long time and can be influenced by anthropogenic action [45].

We found no changes in the Hd index in either species or within the populations when *gp60* was excluded from the CS. However, we did observe lower Pi, S, and K values when this marker was excluded. This result could be expected since the *gp60* sequence represents around one-third of the CS alignment. Despite the nucleotide diversity changes, no alterations in the inter- and intraspecies data comparisons were detected, providing evidence that markers other than *gp60*, such as those used in this work, can be used for genetic diversity analysis [24, 46, 47].

We highlight the results obtained with the IbA10G2 subtype samples, the most common *C*. *hominis* subtype worldwide. This subtype was separated into 8 different MLG, according to our results. These results support the suitability of the six markers for improved genotyping of *C*. *hominis* IbA10G2, fulfilling the need for epidemiological markers with greater discriminatory power than that observed with *gp60*. Other authors have evaluated the potential use of MLST for assessing the intra-genetic diversity of *C*. *hominis gp60* subtypes. Beser et al. using a panel of nine loci detected 10 different genetic profiles in IbA10G2 samples, with all the isolates from outbreaks or clusters belonging to the same profile [48]. They demonstrated a great value of MLST based methods in the outbreaks and transmission dynamics studies of clinically important *Cryptosporidium* subtypes.

In Colombia, only one multi-locus analysis work has been published, which was based on the MLFT approach in the study of cattle samples [12]. Avendaño et al. found three *C*. *parvum gp60* subtypes that were also identified in our study (IIaA17G4R1, IIaA20G6R1, and IIaA18G5R1), providing evidence of the zoonotic potential of this species and the importance of cattle in cryptosporidiosis transmission in Colombia [12]. In the MLFT analysis, ten genetic markers were included, five of which were also used in our work (*CP47*, *gp60*, *ML2*, *MSC6–7*, and *TP14*). The *ML2* marker was the most polymorphic one, with the highest number of alleles. In our study, this marker was excluded from the unilocus and multilocus analysis since it could not be amplified in all the samples evaluated. Other results obtained in the MLFT study from cattle in Colombia established *TP14* as the only marker with a monomorphic behavior [12], which differs from our results, where a polymorphism was identified. However, this could be associated with different analysis strategies and sample origins.

Inference of evolutionary relationships between *C*. *hominis* and *C*. *parvum*, based on MLG, is presented in this study. Three internal subgroups were observed within Group 2, one labeled as 2.1 that included the *C*. *hominis* MLG classified within the IbA10G2 *gp60* subtype; and the MLG 9 and 11, corresponding to the *C*. *hominis* samples classified in the Id and Ie *gp60* subtype family, respectively. In this subgroup, MLG1 was identified as the common ancestor for the MLG 2, 11, and 12, showing that the parasites of the allelic family Ib and Ie have the same common ancestor. These results agree with other reports in the literature that show that Ie and Ib allelic families are evolutionarily and phylogenetically closer [45].

Regarding one sample classified as IbA10G2 subtype and belonging to the MLG7, it was evolutionarily distant from the other MLG obtained from the samples with the same subtype. An event of geographic segregation could explain this result since this was the only sample of the IbA10G2 subtype identified in children from Santander province; the other samples were patients from Antioquia province. Additionally, our results show that MLG3, 4, and 8 (*C*. *hominis* Ia subtype family) were evolutionarily close to those of the *C*. *parvum* MLG1* *(*IIc subtype family), which has been described in the literature [45]. *Cryptosporidium parvum* IIc subtype has been associated with an anthroponotic transmission [45, 49, 50], and results from a comparative genomic study carried out by Nader et al. demonstrated that it shares loci with *C*. *hominis*, with high mutation levels in sub telomeric genes, where MEDLE proteins and insulin-like proteases are located [51, 52].

To our knowledge, this is the first study of the genetic variability of parasites of the *Cryptosporidium* genus in human samples based on MLST in Colombia. Diversity values obtained in this work reflect changes in the genetic composition of *Cryptosporidium* parasites identified in Colombian patients, which could be influenced by different evolutionary events. These phenomena should be studied in greater detail with a larger sample size and different host origin and infection sources, so that associations that describe parasite behavior can be established in the country, which, in turn, could be connected to the eco-epidemiological and sanitary conditions of our population. Finally, national reference laboratories which play a central role in the detection, monitoring, and outbreak studies of infectious diseases, such as cryptosporidiosis, should be responsible for standardizing these methodological strategies, having an impact on epidemiological surveillance systems.

## Supporting information

S1 File Supporting information contains all the supporting tables. (DOCX)
