# Supplementary material for: Multilocus Sequence Typing helps understand the genetic diversity of Cryptosporidium hominis and Cryptosporidium parvum isolated from Colombian patients
Source: PLoS One. 2022 Jul 8;17(7):e0270995. doi: 10.1371/journal.pone.0270995 (PMC9269747; doi:10.1371/journal.pone.0270995)
Supplement: S1 File — (DOCX) [file pone.0270995.s001.docx]

**SUPPLEMENTARY MATERIAL**

**S1 Table. *Cryptosporidium* species and GenBank accession number of 18S rRNA gene sequences used in the dataset for species identification**

| *Cryptosporidium* species | GenBank accession number |
| --- | --- |
| *C. hominis* | AF108865 |
| *C. parvum* | AF112571 |
| *C. cuniculus* | AY120901 |
| *C. meleagridis* | AF112574 |
| *C. ubiquitum* | KT922236 |
| *C. suis* | AF115377 |
| *C. fayeri* | AF108860 |
| *C. felis* | AF108862 |
| *C. canis* | AF112576 |
| *C. bovis* | AY741305 |
| *C. scrofarum* | KF597534 |
| *C. baileyi* | L19068 |
| *C. serpentis* | AF093499 |
| *C. andersoni* | AF093496 |
| *C. muris* | B089284 |
| *C. fragile* | EU162751 |

**S2 Table. *Cryptosporidium* species and GenBank accession number of *gp60 gene* sequences used in the dataset for subtype identification**

| *Cryptosporidium* species | GenBank accession number |
| --- | --- |
| *C. hominis* | AF164502 |
|  | MG694239.1 |
|  | EU161649.1 |
|  | DQ665694 |
|  | KJ910023 |
|  | LT556067 |
|  | KY990911 |
|  | KY990908 |
|  | KY990897 |
|  | FJ707315 |
|  | HQ389257.1 |
|  | AY262031 |
|  | DQ665688 |
|  | MH161561.1 |
|  | FJ707312 |
|  | FJ707314 |
|  | KY990911 |
|  | EF035554.1 |
|  | JX088403.1 |
|  | DQ665692 |
|  | KY990903 |
|  | FJ707316 |
|  | AY738184 |
|  | AF440638 |
|  | FJ153244 |
|  | EF208067 |
|  | FJ971716 |
|  | HM234173 |
|  | JF681174 |
| *C. parvum* | AY262034 |
|  | DQ192501 |
|  | JF727799 |
|  | MF142037.1 |
|  | JQ362495.1 |
|  | EF576961.1 |
|  | JF727759 |
|  | JF727805 |
|  | MF142040 |
|  | MF142041 |
|  | AF402285 |
|  | AF164491 |
|  | AY738194 |
|  | AY382675 |
|  | AY738188 |
|  | AY873780 |
|  | AY873781 |
|  | AY873782 |
| *C. meleagridis* | AF401499 |
|  | AF401500 |
|  | JQ349260 |
|  | JQ349259 |
|  | JQ349257 |
|  | JQ349258 |
|  | JX878610 |
|  | JX878611 |
|  | JX878614 |
|  | JX878613 |
|  | AB539721 |
|  | KJ210619 |
|  | KJ210608 |
|  | AB539718 |
|  | KJ210605 |
|  | KJ210620 |
|  | JF691563 |
|  | JX416370 |
|  | AF401501 |
|  | KJ210613 |
|  | AB539720 |
|  | KJ210618 |
|  | KJ210610 |
|  | KJ210616 |
|  | AB539719 |
|  | KJ210612 |
|  | KJ210609 |
|  | KJ210606 |
|  | AF401498 |
|  | JQ796094 |
|  | JF691562 |
|  | JQ796093 |

**S3 Table. GenBank access codes for each of the study sequences**

| **Sample** | **Species** | **18S rRNA Code** | **gp60 Code** | **CP47 Code** | **MS5 Code** | **MS9 Code** | **MSC6-7 Code** | **TP14**  **Code** |
| --- | --- | --- | --- | --- | --- | --- | --- | --- |
| NA1_Ch | *C. hominis* | MN661181 | MN661176 | MT292558 | MT152383 | ND | MT113934 | MT240800 |
| NA2_Ch | *C. hominis* | MN661182 | MN661177 | MT292559 | MT152384 | MT009633 | MT113935 | MT118815 |
| NA3_Cm | *C. meleagridis* | MN661187 | MN746323 | ND | ND | ND | ND | ND |
| NA4_Ch | *C. hominis* | MN661183 | MN661178 | MT292560 | MT152385 | MT009634 | MT113936 | MT118816 |
| NA5_Ch | *C. hominis* | MN661184 | MN661179 | MT292561 | MT152386 | ND | MT113937 | MT240801 |
| NA6_Ch | *C. hominis* | MN661185 | MN661180 | MT304857 | MT252991 | MT009635 | MT025008 | MT118817 |
| NA7_Ch | *C. hominis* | MN661186 | MN688137 | MT304858 | MT223181 | MT009636 | MT025009 | MT240802 |
| NA8_Ch | *C. hominis* | MT648487 | MN984300 | MT292563 | MT152387 | MT009637 | MT113938 | MT118818 |
| NA9_Ch | *C. hominis* | MT648488 | MN984301 | MT292565 | MT162478 | MT009638 | MT025010 | MT118819 |
| NA10_Ch | *C. hominis* | MT648489 | MN984302 | MT292566 | MT162479 | MT009639 | MT025011 | MT118820 |
| NS1_Cf | *C. felis* | MT648500 | ND | ND | ND | ND | ND | ND |
| NS2_Cp | *C. parvum* | MT648477 | MT009624 | MT341584 | MT180459 | MT025000 | MT137333 | MT103364 |
| NS3_Cp | *C. parvum* | MT648478 | MT009625 | MT341585 | MT180460 | MT025001 | MT137334 | MT103365 |
| NS4_Cp | *C. parvum* | MT648479 | MT009628 | MT341586 | MT180461 | MT025002 | MT137335 | MT103366 |
| NS5_Cp | *C. parvum* | MT648480 | MT024996 | MT341587 | MT180462 | MT025003 | MT137336 | MT103367 |
| NS6_Cs | *C. suis* | MT645275 | ND | ND | ND | ND | ND | ND |
| NS7_Cp | *C. parvum* | MT648481 | MT009626 | MT341588 | MT180463 | MT025004 | MT137337 | MT103368 |
| NS8_Ch | *C. hominis* | MT648490 | MN984298 | MT292564 | MT223182 | MT009640 | MT025012 | MT118821 |
| NS9_Ch | *C. hominis* | MT648491 | MT009623 | MT311158 | MT263150 | MT009641 | MT025013 | MT118822 |
| NS10_Ch | *C. hominis* | MT648492 | MT024997 | MT314432 | MT162480 | MT009642 | MT272827 | MT118823 |
| VA1_Ch | *C. hominis* | MT648493 | MN984299 | MT292562 | MT162476 | MT009629 | MT025006 | MT118811 |
| VA2_Cp | *C. parvum* | MT648482 | MT024994 | MT345597 | MT372487 | MT375485 | MT407457 | MT425057 |
| VA3_Cp | *C. parvum* | MT648483 | MT009627 | MT353685 | MT247675 | MT024999 | MT137331 | MT103363 |
| VA4_Ch | *C. hominis* | MT648494 | MT024998 | MT333777 | MT152381 | MT009630 | MT270452 | MT118812 |
| VA5_Cp | *C. parvum* | MT648484 | MT024995 | MT341583 | MT247676 | MT025005 | MT137332 | MT103369 |
| VA6_Ch | *C. hominis* | MT648495 | MN984303 | MT292567 | MT152382 | MT009631 | MT113933 | MT118813 |
| VA7_Ch | *C. hominis* | MT648496 | MN984304 | MT292568 | MT162477 | MT009632 | MT025007 | MT118814 |
| VA8_Cf | *C. felis* | MT648501 | ND | ND | ND | ND | ND | ND |

ND: Not determined**.**

**S4 Table. *Cryptosporidium* genomes selected for the validation strategy for the MLST genetic markers.**

| **Selected genomes from CryptoDB database** | | |
| --- | --- | --- |
| **Query: GenBank reference partial sequences of each marker** | **Subject: CryptoDB genomes included in the study** | **gp60 unilocus subtypes** |
| - CP47 (AF384127.1) - gp60 (AY262031) - ML2 (AF344880.1) - MS5 (JX413499.1) - MS9 (KP172519.1) - MSC6-7 (GU933484.1) - TP14 (KP172516.1) | *C. hominis* UdeA01 | **Ie**A11G3T3 |
|  | *C. hominis* TU502-2012 | **Ib**A10G2 |
|  | *C. parvum* Iowa II | **IIa**A15G2R1 |
|  | *C. meleagridis* UKMEL1 | **IIIb**A22G1R1 |
|  | *C. tyzzeri* UGA55 | **IX**A6 |
| **Selected genomes from NCBI SRA database** | | |
| **Query:** **Complete sequences of each marker from the *C. hominis* UdeA01 genome** | **Subject:** **SRA genomes included in the study** | **gp60 unilocus subtypes** |
|  | *C. hominis* UKH3 (SRX3244036) | **Ib**A10G2 |
|  | *C. hominis* UKH4 (SRX3255852) | **IA**A14R3 |
|  | *C. parvum* UKP2 (SRX3230187) | **IIa**A19G1R2 |
|  | *C. parvum* UKP3 (SRX3259521) | **IIa**A18G2R1 |
|  | *C. parvum* UKP4 (SRX3259624) | **IIa**A15G2R1 |
|  | *C. parvum* UKP5 (SRX3259631) | **IIa**A15G2R1 |
|  | *C. parvum* UKP6 (SRX3259988) | **IIa**A15G2R1 |
|  | *C. parvum* UKP7 (SRX3260007) | **IIa**A17G1R1 |
|  | *C. parvum* UKP8 (SRX3260278) | **IId**A22G1 |
|  | *C. parvum* UKP15 (SRR6813719) | **IIc**A5G3a |

| Genetic marker | Primers | PCR Type | Amplification protocol | Concentration primers  PCR 1/ PCR 2 | Amplicon size (pb) | PCR additive | DNA volume PCR 1/ PCR 2 | % Amplification in 24 samples |
| --- | --- | --- | --- | --- | --- | --- | --- | --- |
| gp60 | F1: ATAGTCTCCGCTGTATTC  R1: GGAAGGAACGATGTATCT  F2: TCCGCTGTATTCTCAGCC  R2: GCAGAGGAACCAGCATC | Nested | 95ºC x 60seg  95ºC x 30seg  55ºC x 30seg  72ºC x 60seg/ 35 cycles  72ºC x 5min | 5μM/ 5μM | 800-850 | BSA | 1μl/2μl | 100 |
| CP47 | F1: GCTTAGATTCTGATATGGATCTAT  R1: AGCTTACTGGTCCTGTATCAGTT  F2: ACCCCAGAAGGCGGACCAAGGTT  R2: GTATCGTGGCGTTCTGAATTATCAA | Nested | 94ºC x 5min  94ºC x 45seg  55ºC x 45seg  72ºC x 60seg/35 cycles  72ºC x 10min | 5μM/ 5μM | 350-600 | BSA and DMSO | 1μl/1μl | 100 |
| ML2 | F1: CAATGTAAGTTTACTTATGATTAT  R1: CGACTATAAAGATGAGAGAAG | Endpoint | 94ºC x 5min  94ºC x 30seg  55ºC x 30seg  72ºC x 60seg/ 40 cycles  72ºC x 7min | 5μM | 175-237 | BSA | 2μl | 37,5 |
| MS5 | F1: GCATGTAGTCGTATCCGGAAC  R1: GTATGCTGGGGAATATAGCCAAG | Endpoint | 95ºC x 5min  95ºC x 50seg  55ºC x 50seg  70ºC x 60seg/35 cycles  72ºC x 10min | 5μM | 181-502 | BSA and DMSO | 3μl | 100 |
| MS9 | F1: TTAGTCGACCTCTTCAACAGTTGG  R1: CAGAATTGGAATCATTTTCTGAAT  F2: GGACTAGAAATAGAGCTTTGGCTGG  R2: GTCTGAGACAGAATCTAGGATCTAC | Nested | 95ºC x 5min  95ºC x 50seg  55ºC x 50seg  65ºC x 60seg/ 35 cycles  72ºC x 7min | 5μM/ 5μM | 303-534 | BSA and DMSO | 1μl/1μl | 92% |
| MSC6-7 | F1: ATTGAACAAACGCCGCAAATGTACA  R1: CGATTATCTCAATATTGGCTGTTATTGC  F2: GCTATTTGCTATCGTCTCACATAACT  R2: CTACTGAATCTGATCTTGCATC | Nested | 94ºC x 5min  94ºC x 45seg  55ºC x 45seg  72ºC x 60seg/35 cycles  72ºC x 10min | 5μM/ 5μM | 455-560 | BSA | 1μl/1μl | 100 |
| TP14 | F1: GAGAAGGAGCAATGGGAGCA  R1: TCCTCCTTTTTGCCCTTGAA  F2: CTAACGTTCACAGCCAACAGTACC  R2: CAATAAAGACCATTATTACCACC | Nested | 95ºC x 3min  95ºC x 50seg  55ºC x 50seg  65ºC x 60seg/ 35 cycles  72ºC x 10min | 5μM/ 5μM | 234-342 | BSA and DMSO | 1μl/2μl | 100 |

**S5 Table. Standardized protocols for amplification of the seven markers selected for MLST analyzes**

**S6 Table. Intra-species and intra-population genetic variability indices**

| ***C. hominis* samples**  **Gene variability indices based on the Consensus Sequence of the six markers used for MLSTs** | | | | | |
| --- | --- | --- | --- | --- | --- |
| Total samples: 14 | **# MLG** | **Hd** | **Pi** | **S** | **K** |
|  | 13 | 0,99 +/- 0,03 | 0,09 +/- 0,05 | 715 | 220,77 +/- 100,63 |
| Children from Antioquia (NA): n7 | 6 | 0,95 +/- 0,096 | 0,08 +/- 0,05 | 459 | 201,81 +/- 98,78 |
| Children from Santander (NS): n3 | 3 | 1,0 +/- 0,27 | 0,15 +/- 0,11 | 520 | 361,67 +/- 216,5 |
| HIV (+) patients from Antioquia (VA): n4 | 4 | 1,0 +/- 0,18 | 0,07+/- 0,04 | 292 | 154,17 +/- 84,6 |
| ***C. hominis* samples**  **Gene variability indices based on the Consensus Sequence excluding *gp60*** | | | | | |
| Total samples (14) | 13 | 0,99 +/- 0,03 | 0,05 +/- 0,02 | 296 | 76,1 +/- 34,9 |
| Children from Antioquia (NA): n7 | 6 | 0,95 +/- 0,09 | 0,04 +/- 0,03 | 183 | 70,7 +/- 34,8 |
| Children from Santander (NS): n3 | 3 | 1,0 +/- 0,3 | 0,07 +/- 0,05 | 158 | 105,67 +/- 63,5 |
| HIV (+) patients from Antioquia (VA): n4 | 4 | 1,0 +/- 0,18 | 0,04 +/- 0,03 | 115 | 65,67 +/- 36,24 |
| ***C. parvum* samples**  **Gene variability indices based on the Consensus Sequence of the six markers used for MLSTs** | | | | | |
| Total samples: 8 | **# MLG** | **Hd** | **Pi** | **S** | **K** |
|  | 8 | 1,0 +/- 0,06 | 0,06 +/- 0,03 | 479 | 128,36 +/- 61,8 |
| Children from Santander (NS): n5 | 5 | 1,0 +/- 0,13 | 0,005 +/- 0,003 | 23 | 10,8 +/- 5,9 |
| HIV positive patients from Antioquia (VA): n3 | 3 | 1,0 +/- 0,27 | 0,13 +/- 0,09 | 467 | 311,33 +/- 186,4 |
| ***C. parvum* samples**  **Gene variability indices based on the Consensus Sequence excluding *gp60*** | | | | | |
| Total samples (8) | 8 | 1,0 +/- 0,06 | 0,04 +/- 0,02 | 261 | 70,07 +/- 33,9 |
| Children from Santander (NS): n5 | 5 | 1,0 +/- 0,13 | 0,003 +/- 0,002 | 22 | 9,2 +/- 5,1 |
| HIV positive patients from Antioquia (VA): n3 | 3 | 1,0 +/- 0,27 | 0,1 +/- 0,07 | 246 | 164,0 +/- 98,3 |

**Hd**: Haplotype diversity, **Pi:** Nucleotide diversity, **S**: Number of segregating sites, **K:** Nucleotide differences

**S7 Table. Intra-species and inter-population gene Fixation index (Fst)**

| ***C. hominis* samples**  **F_st_ with the Consensus Sequences (CS) of the six markers used for the MLSTs** | | | | |
| --- | --- | --- | --- | --- |
| **Studied population** | **NA (7)** | **NS (3)** | | **VA (4)** |
| Children from Antioquia  (NA): n7 | - |  | |  |
| Children from Santander  (NS): n3 | -0.001 | - | |  |
| HIV (+) patients from Antioquia  (VA): n4 | -0.014 | 0,104  *p = 0,12* | | *-* |
| ***F_st_ C. hominis* with the Consensus Sequences excluding *gp60*** | | | | |
| Children from Antioquia  (NA): n7 | *-* |  | |  |
| Children from Santander  (NS): n3 | 0,04  *p= 0,3* | - | |  |
| HIV (+) patients from Antioquia  (VA): n4 | 0,05  *p= 0,54* | 0,04  *p = 0,2* | | - |
| ***C. parvum* Samples**  **F_st_ with the Consensus Sequences of the six markers used for the MLSTs** | | | | |
| **Clinic populations** | **NS (5)** | | **VA (3)** | |
| Children from Santander  (NS): n5 | - | |  | |
| HIV (+) patients from Antioquia  (VA): n3 | 0,23  *p = 0,018** | | - | |
| ***F_st_ C. parvum* with the Consensus Sequences excluding *gp60*** | | | | |
| Children from Santander  (NS): n5 | - | |  | |
| HIV (+) patients from Antioquia (VA): n3 | 0,24  *p = 0,01 ** | | - | |

* Significant (p ≤ 0.05)
